# *Staphylococcus epidermidis* Controls Opportunistic Pathogens in the Nose, Could It Help to Regulate SARS-CoV-2 (COVID-19) Infection?

**DOI:** 10.3390/life12030341

**Published:** 2022-02-25

**Authors:** Silvestre Ortega-Peña, Sandra Rodríguez-Martínez, Mario E. Cancino-Diaz, Juan C. Cancino-Diaz

**Affiliations:** 1 Laboratorio Tejido Conjuntivo, Centro Nacional de Investigación y Atención de Quemados, Instituto Nacional de Rehabilitación “Luís Guillermo Ibarra Ibarra”, Ciudad de México 14389, Mexico; 2 Laboratorio de Inmunidad Innata, Departamento de Inmunología, Escuela Nacional de Ciencias Biológicas, Instituto Politécnico Nacional, Ciudad de México 11340, Mexico; sandrarodm@yahoo.com.mx (S.R.-M.); mecancinod@gmail.com (M.E.C.-D.); 3 Laboratorio de Inmunomicrobiología, Departamento Microbiología, Escuela Nacional de Ciencias Biológicas, Instituto Politécnico Nacional, Ciudad de México 11340, Mexico

**Keywords:** respiratory microbiome, dysbiosis, SARS-CoV-2 infection, COVID-19, *Staphylococcus epidermidis*, probiotics

## Abstract

*Staphylococcus epidermidis* is more abundant in the anterior nares than internal parts of the nose, but its relative abundance changes along with age; it is more abundant in adolescents than in children and adults. Various studies have shown that *S. epidermidis* is the guardian of the nasal cavity because it prevents the colonization and infection of respiratory pathogens (bacteria and viruses) through the secretion of antimicrobial molecules and inhibitors of biofilm formation, occupying the space of the membrane mucosa and through the stimulation of the host’s innate and adaptive immunity. There is a strong relationship between the low number of *S. epidermidis* in the nasal cavity and the increased risk of serious respiratory infections. The direct application of *S. epidermidis* into the nasal cavity could be an effective therapeutic strategy to prevent respiratory infections and to restore nasal cavity homeostasis. This review shows the mechanisms that *S. epidermidis* uses to eliminate respiratory pathogens from the nasal cavity, also *S. epidermidis* is proposed to be used as a probiotic to prevent the development of COVID-19 because *S. epidermidis* induces the production of interferon type I and III and decreases the expression of the entry receptors of SARS-CoV-2 (ACE2 and TMPRSS2) in the nasal epithelial cells.

## 1. Introduction

Respiratory tract infections (RTIs) cause high morbidity and mortality. Viruses (influenza, parainfluenza, respiratory syncytial virus, coronavirus, human metapneumovirus and rhinovirus) and bacteria represent the most common cause of respiratory infections in children, the elderly, and immunocompromised patients [1,2,3]. RTIs contribute annually to substantial morbidity and mortality around the world that have eventually led to pandemics that have destroyed societies and economies [4,5] such as Spanish influenza, 1918; Asian flu, 1957; Hong Kong flu, 1968; Severe Acute Respiratory Syndrome (SARS), 2002; Middle East Respiratory Syndrome (MERS), 2004; Influenza A (H1N1), 2009; and recently COVID-19 (SARS-CoV-2), 2019 [5,6,7,8,9,10].

The spectrum of respiratory infections caused by viruses and bacteria is heterogeneous, ranging from mild upper respiratory infections to severe life-threatening lower respiratory infections, including the development of acute lung injury and acute respiratory distress syndrome [11,12]. After a lung infection, some patients may suffer sequelae or develop new pathologies. Viral infections in early life cause acute illness, and in adulthood viral infections may be associated with the development of wheezing and asthma [13]. In COVID-19 it has been described that after the disease some patients develop “post-COVID syndrome”, which includes persistent symptoms that could be related to residual inflammation, organ damage, nonspecific effects of hospitalization or prolonged ventilation (post-COVID syndrome -intensive care), as well as negative impacts on pre-existing health conditions [14].

The susceptibility to suffer RTIs with high severity and to present post-infection disease sequelae depends on the pathogenicity mechanisms of the microbial agent and the intrinsic factors of the host [15]. Regarding microbial factors, there is the ability of respiratory pathogens to evade the host’s innate and adaptive immunity, as well as the ability to alter the inflammatory response [15,16]; and regarding host factors, there are age, sex, morbidities, immaturity or senescence of the immune system, genetic variations, and microbial composition of the respiratory tract, among others [1,15,16].

The human upper respiratory tract (URT) hosts a well-documented bacterial community, or microbiota, that resides in the nasal cavity and nasopharynx. The URT microbiota acts as a guardian that acts against respiratory pathogens, in addition, the microbiota is involved in the maturation and maintenance of homeostasis of respiratory physiology and immunity [17,18,19]. The imbalance in the number or type of microbial community (dysbiosis) in the URT increases the host’s susceptibility to colonization with respiratory pathogens (viruses and bacteria) and increases the risk of suffering RTIs both in the upper and lower tract [15,16,19,20,21]. Dysbiosis in the URT can be caused by various factors, the most common being: age, type of colonization of the URT at birth, prolonged use of antibiotics, chronic inflammatory diseases, etc. [22,23,24,25] Reversing dysbiosis in the URT is important, mainly the microbiota of the nasal cavity since it is one of the most common routes through which respiratory pathogens enter [18]. A therapeutic strategy to protect the host against respiratory pathogens is the nasal application or oral administration of probiotic bacteria [26,27,28]. Probiotics are “living microorganisms” that when administered in adequate amounts confer benefits for the health of the host [26].

*Staphylococcus epidermidis* is one of the most abundant commensal bacteria in human skin and mucosa. Furthermore, *S. epidermidis* is the most abundant species in the microbiota of the human nasal cavity, but its abundance in the nose changes throughout the life of humans [22,29,30,31]. It is now known that *S. epidermidis* has the ability to regulate and train the immune system of human skin and apparently does so in the nasal cavity as well, as it can protect against colonization by pathogenic bacteria and respiratory viruses [32]. Although little has been studied on the use of *S. epidermidis* as a probiotic in the respiratory tract, this review aims to demonstrate the importance of *S. epidermidis* in the control and inhibition of respiratory tract pathogens and to establish a possible role on the SARS-CoV-2 virus causing COVID-19.

## 2. Overview of the Nasal Cavity

The respiratory system is a set of complex organs divided into the upper respiratory tract, which includes the nasal cavity, pharynx, and larynx, and the lower respiratory tract, which includes the trachea, bronchi, bronchioles, and alveoli. With respect to the nasal cavity, it is divided anatomically into the anterior nostril, and the inferior, middle, and superior meatus, which are separated by three nasal turbinates [19]. Other anatomical structures that communicate with the nasal cavity are the sphenoethmoidal recess and nasopharynx. The anterior nares are the entrance to the nasal cavity and lead directly to the vestibule. The middle meatus is adjacent to the vestibule, and the nasopharynx connects to the throat. The sphenoethmoidal recess is located within the posterior portion of the nasal cavity. The olfactory region is located at the ceiling of the nasal cavity. The frontal and sphenoid sinuses are located within the facial skeleton [19] Both the nasal cavity and the turbinates perform important physiological functions such as filtering, heating and humidifying the inhaled air. Other functions are olfactory detection and defense against respiratory pathogens [19].

Each anatomical area has a specific function and at the same time a different cellular composition, however, the common component is the epithelial cells that are present throughout the airway. Within these epithelial cells, the main cells that make up the respiratory tract are ciliated cells and secretory cells (club and goblet cells) that are specialized for mucociliarity that helps eliminate airborne infectious particles and pathogens that enter the respiratory tract. Regarding the epithelial cells that line the nasal cavity, they can be of different types: the anterior naris starts with non-keratinized skin-like epithelium (1), changing into stratified squamous epithelial cells without microvilli (2), followed by transitional epithelium with short microvilli (3), before transition into the middle meatus with its pseudostratified columnar epithelium (4 and 5, middle meatus) [19]. Secretory cells (club and goblet cells) mainly secrete mucus, which has the function of trapping particles and antigens carried into the respiratory system during inhalation. Mucus that has trapped particles or pathogens can bind to secretory IgA dimers, which prevents attachment of pathogens to host epithelium, thus hindering invasion [33]. Cilia of epithelial cells within the nasal cavity also function to propel mucus away from the lungs in an attempt to expel trapped pathogens from the body [34]. Sinonasal epithelial cells generate and secrete antimicrobial compounds to directly counteract pathogens. These compounds have various antibacterial, antifungal, and antiviral effects, and include proteins such as lysozyme, lactoferrin, defensins, and cathelicidins, as well as reactive oxygen and nitrogen species, e.g., nitric oxide (NO) [35]. Ciliated cells and secretory cells are the components of the physical epithelial cell barrier, which by means of mucociliary clearance, and antimicrobial compound secretion play pivotal innate immune roles in defending the sinonasal cavity from infection [35].

In each anatomical site of the respiratory tract, there is the presence of progenitor cells, such as basal cells in the airway and AT2 cells in the alveoli. These cells are responsible for epithelial regeneration under homeostatic conditions or in cases of tissue damage [36]. Other cells identified in the respiratory tract are suprabasal cells with intermediate function between basal cells and club cells, and deutorosomal cells that are the precursors of ciliated cells specialized in the elimination or clearance of mucus and debris. There are other cells that have been recently identified; the mucous-ciliated cells with an intermediate stage of differentiation between goblet and ciliated cells; ionocytes, which are cells that transport ions, regulate fluids and pH; pulmonary neuroendocrine cells that participate in the sensory part; tuft/brush cells that are immune and taste sensor cells that produce leukotriene and links to type 2 immunity; and finally the Hillock cells, which are transient between basal and secretory cells, located in stratifications, with non-ciliated structures and high cell turnover with a function of squamous barrier and in immunomodulation [37,38].

Like any other organ, the presence of an immune response system is essential for its homeostasis, and in the case of the respiratory tract, it is not an exception. Airway cells have immune functions such as secretory epithelial cells that produce antimicrobial mediators and mucins such as MUC5AC and MUC5B that contribute to the first defense barrier of the host on the epithelial surface of the airway [39]. Another example is the secreted IgA (SigA) produced by sub-epithelial plasma cells transported to the apical surface of the cells of the airway epithelium by means of the polymeric immunoglobulin receptor (pIgR), this immunoglobulin prevents the adherence of microorganisms in a process called “immune exclusion” [33]. On the other hand, epithelial cells have pattern recognition receptors, such as Toll-like receptors, to quickly detect the presence of antigens or microorganisms and initiate an immune response; in addition, these cells have cytokine receptors, such as TNFR1, to respond to the signals produced by immune cells such as airway macrophages [40]. 

The immune cells present in the nasal cavity with the highest abundance are macrophages, located in the lumen of the airway [41]. Airway-resident macrophages interact with epithelial cells to maintain the state of homeostasis and respond to inhaled pathogens, as well as tissue repair [42]. Furthermore, also present are the effective adaptive T cells that participate in the immune response against allergy, pathogens, and antigens. These T cells are a population of tissue-resident memory T (TRM) cells [43] with characteristics that differ from circulating effector memory T cells and express adhesion molecules that promote their retention within mucosal tissue [44]. TRM cells can be classified into CD4 + and CD8 + TRM cells that respond against antigens present in the airway to generate a rapid and subsequent immune response [45]. Another type of T lymphocytic cells that connect the innate immune response with the adaptive immune response are innate like T lymphocytes called mucosal-associated invariant T (MAIT) cells, these cells constitute approximately 4% of the total T cells in the human airway wall [46]. MAIT cells express a semi-invariant αβ T cell receptor (TCR) that allows the recognition of riboflavin metabolites (vitamin B2) biosynthesis derived from bacteria and yeasts and presented by MHC-related protein 1 (MR1) [47]. Activated MAIT cells rapidly generate pro-inflammatory cytokines for protection against respiratory pathogens [48], however, these cells have other functions such as maintaining post-infection barrier integrity and healing [49].

## 3. Microbiota of the Nasal Cavity

It is important to mention that the microbes that live in the nasal cavity are subjected to a variety of stress conditions that they have to counteract in order to survive and persist, since the nasal cavity is a nutrient-poor environment, with an acid and saline condition [50]. In addition, the microenvironment of the nasal cavity is different in each anatomical location, for example, the anterior nares (nostrils), is the most acidic environment with high salinity, and therefore, this area is the most difficult for the survival of microbes [51]. Adjacent to the anterior nares is the middle meatus, the largest portion of the nose that encompasses a network of bone and mucosa where the mucin-secreting goblet cells, responsible for the production of mucus, restrict microbial growth. The remaining portion of the nasal cavity contains the classical upper airway ciliated pseudostratified and columnar epithelial cells, that aid the movement of airborne particles during their passage through the nose [51]. This posterior region of the nasal cavity also includes the sphenoethmoidal recess, which allows the drainage of the sphenoidal and ethmoid sinuses.

The nasal cavity is directly connected to the external environment and in direct contact with various microorganisms, and through inhalation, it can internalize a great diversity of microbes, fungal spores and different environmental pollutants, however, despite this great variety of microorganisms, there is a clear predominance of different genera or species that remain in the upper respiratory tract of healthy adults [52]. The study of the nasal microbiota has been performed by taking samples from the different parts of the nasal cavity of healthy adults. The microbiota of the anatomical area of the anterior nose is mainly composed of Actinobacteria, Firmicutes, and Proteobacteria [53]. About the bacterial genera in the anatomical zone, the anterior nares of 236 healthy adults have *Staphylococcus*, *Cutibacterium* (before named *Propionibacterium*), *Corynebacterium*, and *Moraxella* [54]. In the anatomical zone of the middle meatus of healthy adults, the most abundant are *Staphylococcus aureus* (*S. aureus*), *S. epidermidis*, and *Cutibacterium acnes* [55]. The genera *Streptococcus*, *Prevotella*, *Veillonella*, and *Haemophilus* are the most abundant in the anatomical area of the throat [56].

Through high-throughput sequencing, it has been shown that the microbial community in the nasal cavity changes throughout the life of an individual depending also on the anatomical location, however, over time the microbiota becomes stabilized [19,57]. For example, in infants, the initial nasal microbiota is similar to the mother’s vaginal and skin microbiota [58]. In healthy adults, the differences in microbial communities are linked to the anatomical location within the nasal cavity. In the anterior nares, middle meatus, and sphenoethmoidal recess reveal different microbial communities at each site [59], in the anterior nares it is mainly composed of Actinobacteria and Firmicutes [57,59], in contrast, similar microbial communities are present in the middle meatus and the sphenoethmoidal recess and contained an enriched amount of Proteobacteria compared to the anterior nares [59]. 

At all sites in the nasal cavity, Actinobacteria is the most predominant and is strikingly present throughout all stages of life [19,57]. *Corynebacterium* and *Cutibacterium* are the most abundant and prevalent species of the genus Actinobacteria in the nasal cavity [53,57,60]. In general, the high abundance of *Corynebacterium* in the nasal cavity is associated with increased stability of the nasal microbiota and decreased risk for respiratory infections, especially in early life [61]; in newborns (before six weeks old) there is a high abundance of *Corynebacterium* in the anterior nares and it is correlated with a decreased risk of rhinitis [61]. In contrast, *Cutibacterium* is more enriched in the nasal cavity of adolescents and is correlated with the onset of acne development [62]. After Actinobacteria, the Firmicutes are the most abundant phylum present in the nasal microbiota [59], including *Staphylococcus*, *Streptococcus*, and *Dolosigranulum*. For example, the *S. epidermidis* commensal species colonizes nearly 100% of individuals early in life, it persists within the nasal cavity and surrounding skin and is associated with a stable nasal microbiota [63]. Proteobacteria such as *Moraxella* and *Haemophilus* can colonize the nasal cavity at similar levels to those of Actinobacteria and Firmicutes in early childhood but then decrease over time toward adulthood [64]; they then remain at a constant albeit low abundance, especially in the anterior nares.

According to the analysis of nasal microbiota by *16s rRNA* gene sequencing and microbial culture in healthy individuals of different age groups (5.45 + 0.50-year-old children (n = 155), 19.47 + 0.73-year-old young adults (n = 171), and elderly 82.50 + 8.29 years of age (n = 141) *S. epidermidis* is predominant over other species (*S. aureus, Moraxella catarrhalis, Corynebacterium propinquum,* and *Corynebacterium pseudodiphteriticum*), being significantly more pronounced in young adults and in lesser extent in the elderly and children [22]. *S. epidermidis* has been given the function of maturing the microbiota of the nasal cavity in young adults, since *S. epidermidis* induces the production of antimicrobial peptides (AMP) in nasal epithelial cells to eliminate competitive pathogens, besides it is resistant to AMPs through biofilm formation, thus indicating symbiotic cooperation between this bacterium and the host’s innate nasal response [22]. For this reason, the decrease in *S. epidermidis* increases the susceptibility of the nostrils to colonize with respiratory pathogens and increases the risk of RTIs in children and adults [22,65,66]. However, age is not the only factor associated with a reduction in the abundance of *S. epidermidis* in the nostrils, also chronic inflammatory diseases of the nose such as granulomatosis with polyangiitis (GPA; Wegener’s), sinusitis, and polyposis influence the decrease in *S. epidermidis* in the nasal cavity [67], therefore, in these patients also increases the risk of RTIs [68].

**Figure 1:**
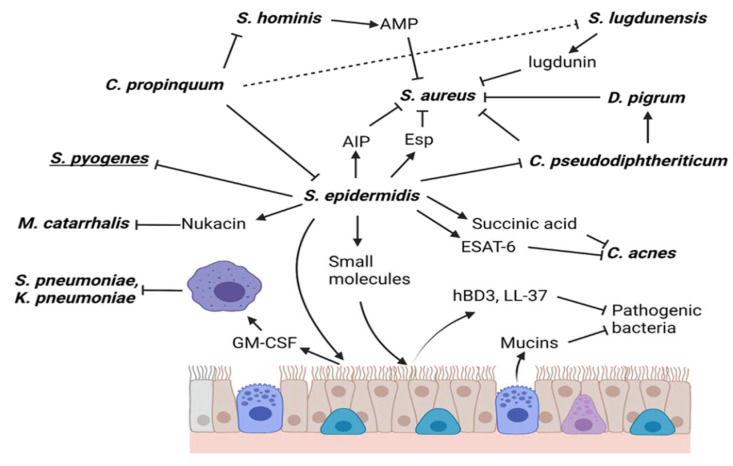
Role of *Staphylococcus epidermidis* in the nasal cavity. *S. epidermidis* has several functions in the nasal cavity, one of them is to inhibit the colonization of *Staphylococcus aureus* through the production of Esp protease and it can also inhibit the System Quorum Sensing of *S. aureus* through the secretion of its Autoinducing Peptides (AIPs). *S. epidermidis* inhibits *Cutibacterium acnes* (*C. acnes*) by the production of succinic acid and by the presence of the ESAT-6 secretion system. Nukacin production inhibits *Moraxella catarrhalis* and *Streptococcus pyogenes* by an unknown mechanism. On the other hand, *S. epidermidis* can activate the innate immune response by producing small molecules that stimulate epithelial cells to produce antimicrobial compounds and cytokines that activate macrophages. Other bacteria can interact to promote or inhibit *S. aureus*.

Because *S. epidermidis* is one of the most abundant bacteria in the nasal cavity and as it has the ability to prevent or attenuate respiratory tract infections caused by respiratory pathogens (mainly viruses and bacteria), in the following topics we will address the mechanisms by which *S. epidermidis* prevents colonization of the nostrils with respiratory pathogens (Figure 1) mainly *S. aureus, D. pigrum, M. catarrhalis, Streptococcus pneumoniae*, *Klebsiella pneumoniae* and influenza A and B viruses. The fact that the nasal application of *S. epidermidis* prevents or attenuates RTIs, both in the upper and lower respiratory tracts, will also be addressed.

## 4. Generalities and Genetic Characteristics of *S. epidermidis*

*S. epidermidis* has two lifestyles: a commensal form and an infectious form. As a commensal form, *S. epidermidis* inhabits the skin and mucosa of humans and other mammals; as infectious form, it is considered an opportunistic pathogen where it uses an infective mechanism through biofilms developed in medical devices, and through this route it can infect the bloodstream, mainly in immunocompromised patients, being the second most commonly isolated opportunistic pathogen [29,30,31]. For the reason that the skin is in direct contact with the environment (temperature changes, acidity, low nutrients, salinity, contact with pathogens, etc.), *S. epidermidis* has the ability to adapt and colonize surfaces with different physicochemical characteristics [30,31]. 

Due to the ability of *S. epidermidis* to adapt to changing environments, there is currently great interest in understanding its genome. The complete genome sequence analysis performed on *S. epidermidis* isolated from different sources (commensal and infection) showed that the bacterium has an open pan-genome with 80% core genes and 20% variable genes. The variable genome is characterized by the abundance of transposable elements, transcription factors and transporters [29,30,31,69], indicating a great genetic diversity among the different strains or isolates, that’s why it has been seen that in the same subject and in the same site in your body there is a genetic variety among *S. epidermidis*. This high genetic variation makes the analysis of *S. epidermidis*’ genetics difficult because it generates different physiological and biochemical behaviors [29,30,31,69]. Despite this genetic variation, it has been possible to distinguish commensal isolates from infective isolates mainly into two phylogenetic groups: one group that contains the majority of commensal and infective isolates, and the other that mainly comprises commensal isolates [69,70]. The characteristic of its pan-open genome partly explains the ability of *S. epidermidis* to adapt and colonize different ecological niches due to the acquisition of new genetic sequences integrated into its genome [32,69,70].

## 5. *S. epidermidis* Directly or Indirectly Kills Respiratory Bacteria

### 5.1. Staphylococcus aureus

*S. epidermidis* limits the colonization, growth, and expression of virulence factors, as well as mediates the direct or indirect death of potentially pathogenic bacteria of the host’s nasal cavity, for example, the opportunistic pathogen methicillin-resistant *S. aureus* (MRSA) [71]. Colonization of the nostrils with MRSA predisposes the host to infections such as pneumonia and also facilitates the transmission of the pathogen to susceptible individuals [71]. The removal of MRSA from the nostrils with antibiotics has become challenging because the bacteria have developed resistance to different families of antibiotics [72]. One strategy that may be effective in preventing and eliminating MRSA from the nares is the intranasal application of *S. epidermidis* [73]. The mechanisms by which *S. epidermidis* eliminates *S. aureus* and other opportunistic pathogens from the nasal cavity are: secretion of enzymes, molecules with antibiofilm activity, Quroum Sensing regulators, bacteriocins, or stimulation of the immune response of the host, mainly from innate immunity [71].

In general, *S. aureus* initiates the colonization of the host’s nasal cavity through the proteins that decorate its bacterial cell wall, which are called cell wall-anchored proteins (CWA) [74]. *S. aureus* CWAs adhere to host molecules (fibrin, fibrinogen, collagen, etc.) [74]. After the initial adhesion of *S. aureus*, more bacterial cells adhere and form aggregates to form biofilms [74]. Biofilms resist the action of antimicrobial molecules (antiseptics, antibiotics, etc.) and the activity of cells of the innate and adaptive immunity of the host, causing an infective development [75].

*S. epidermidis* eliminates *S. aureus* by the production and secretion of the serine protease Esp (MW 27 kDa) that degrades the CWA of *S. aureus* by proteolysis avoiding the formation of biofilms (adhesion and aggregation) [76,77]. In healthy subjects, it was shown that the nasal cavities of some individuals are colonized with both species of *S. aureus* and *S. epidermidis*, and the rest of the population are colonized only by *S. epidermidis* [78]. In cell-free conditioned media (CFCM) derived from *S. epidermidis* isolated from subjects that are not colonized with *S. aureus*, the presence of Esp protease was detected, and it was demonstrated that the use of these CFCMs inhibits the formation of biofilm of *S. aureus* in a dose-dependent manner by [78]. On the other hand, in this same study, it was demonstrated that *S. epidermidis* strains that secrete Esp in the nostrils of healthy subjects cause the absence of *S. aureus* [78]. This same effect was observed when inoculating recombinant Esp or Esp-producing strains into the nostrils of healthy subjects [78]. This shows that *S. epidermidis*, which produces Esp, protect against colonization and biofilm formation of *S. aureus*.

*S. epidermidis* strains obtained from the nasal cavity of healthy individuals also secrete small molecules (<10 kDa) with *S. aureus* antibiofilm activity, even against methicillin-sensitive or methicillin-resistant biofilm-producing *S. aureus* [79]. In addition, these small molecules can break down biofilms of both protein and carbohydrate composition such as polysaccharide intercellular adhesin/poly -(1-6) -N-acetylglucosamine (PIA/PNAG) from *S. aureus*, and this effect is synergistic when used with oxacillin, however, these small molecules have no effect on the viability of *S. aureus* [79]. The mechanism of action of these small molecules is by the induction of the *icaR* gene expression in *S. aureus*, which is an important negative regulator for the expression of the *icaADBC* operon, that codes for the genes responsible for the synthesis of PIA/PNAG, the main component of carbohydrate phenotype biofilms. Other mechanisms of action of small molecules are: the activation of the transcriptional regulators *rsp*, and AraC-type that inhibit the adhesion and formation of biofilms; other mechanism is the reduction in the expression of other genes involved in biofilm formation such as *sasG*, virulence genes (*spaA*), and *agr* system genes (*agrA, agrB*, and *hld*). The Agr system regulates the expression of genes encoding CWA and ClpC proteins, where ClpC is an ATPase required for stress tolerance and biofilm formation [80]. The biochemical nature of these small molecules produced by *S. epidermidis* remains unknown since they do not lose their functionality under treatment with proteinase K, trypsin, sodium periodate, a cocktail of protease inhibitors or heating [80], the only known fact is that they are molecules of a size between 3 and 10 kDa with hydrophobic nature [79].

The staphylococcal Agr system is a Quorum Sensing system of cell–cell communication, dependent on the bacterial growth phase and bacterial density. Agr controls the expression of virulence factors, biofilm-associated molecules, and the interaction with the host’s innate immune system, therefore it is essential during host colonization and infection [80,81]. Agr responds to an extracellular signal coordinated by the autoinducing peptide (AIP) secreted by *Staphylococcus* [80,81]. Four subgroups of AIP have been identified in *S. aureus* (AIP-SAU 1-4), and three in *S. epidermidis* (AIP-SE1 to AIP-SE3), and each one activates its own Agr receptor [78,79]. The activation of Agr can be competitively inhibited with the different AIPs within the same species or with the AIPs from other *Staphylococcus*, resulting in the inhibition of the expression of molecules associated with biofilms and virulence of *Staphylococcus* [82,83,84].

Otto M, et al. (1999) [82], demonstrated with in vitro experiments that treatment of *S. aureus* with a synthetic AIP from *S. epidermidis* competitively inhibited the Agr system of *S. aureus* and repressed the secretion of δ-toxin and α-toxin, which are the two most important virulence factors of *S. aureus*. Until now, the strategy of inoculating the nasal cavity with *S. epidermidis* able to secrete different AIPs to inhibit *S. aureus*’ Agr system has not been tested, nor it has been tried intranasally inoculating the different *S. epidermidis*’ AIPs as a possible strategy to prevent UTR’s infections caused by *S. aureus*. This proposal is supported because in the skin the *S. epidermidis*’ AIPs have an effect of avoiding colonization of *S. aureus* [83].

On the other hand, we need to highlight that *S. epidermidis* is not the only bacterium that has activity against *S. aureus*; other bacteria that inhabit the nasal cavity can also do so, as in the case of coagulase-negative *Staphylococcus* (CoNS) *S. hominis* and *S. lugdunensis* which, through the production of antimicrobial peptides and the compound lugdunin, respectively, inhibit the growth of *S. aureus* [85,86]. These bacteria, including *S. epidermidis*, can be inhibited by *C. propinquum* through the production of siderophores to sequester iron [87]. *C. pseudodiphtheriticum, Corynebacterium striatum* [88], and *Dolosigranulum pigrum* can inhibit *S. aureus*, and *C. pseudodiphtheriticum* has a growth-promoting effect over *D. pigrum* [89]. Related to opportunistic pathogens, *S. pneumoniae* inhibits the growth of *S. aureus* through the production of reactive oxygen species (ROS) and hydrogen peroxide production [90,91]. On the contrary, there are bacteria present in the nasal cavity that benefit the growth of *S. aureus*, such as *Corynebacterium accolens* which, by releasing free fatty acids, helps its growth [92], as well as *C. acnes* that by the Christie-Atkins-Munch Petersen factor (CAMP) promotes the development of *S. aureus* [93]. In the case of *C. accolens*, it helps to inhibit *S. pneumoniae* through the LipS1 molecule and free fatty acids [92]. The importance of inhibiting or attenuating *S. aureus* in the nasal cavity lies in the ability of *S. aureus* to lyse red blood cells; lysed cells release hemin and NAD into the extracellular milieu [94], these compounds can support and enhance the colonization of *H. influenzae* generating a pathogenic symbiosis between these two bacterial species. In contrast, *S. pneumoniae* can negatively impact *H. influenzae* by the production of hydrogen peroxide [90] and the secretion of the neuraminidase NanA that removes sialic groups from *H. influenzae* lipooligosaccharides (LOSs), which may negatively impact *H. influenzae* attachment to epithelial cells and thus reduce colonization potential [95]. *S. epidermidis* and the other bacteria above mentioned that inhibit *S. aureus* help to prevent the association between *S. aureus* and *H. influenzae*.

### 5.2. Corynebacterium pseudodiphtheriticum

*C. pseudodiphtheriticum* has the ability to prevent colonization and eradication of *S. aureus* from the nasal cavity since individuals colonized with *C. pseudodiphtheriticum* have a very low probability of nasal colonization by *S. aureus* [59]. In vitro, *C. pseudodiphtheriticum* can inhibit the growth of *S. aureus*, and inoculated into the nasal cavity it leads to the eradication of *S. aureus* in healthy volunteers [96] since *C. pseudodiphtheriticum* releases a diffusible compound with bactericidal activity against *S. aureus* [97]. Interestingly, while *C. pseudodiphtheriticum* negatively impacts *S. aureus* viability, *C. pseudodiphtheriticum* does not impact the viability of *S. epidermidis*, which is closely related to *S. aureus*. Instead, at least one study demonstrated that *S. epidermidis* inhibits the growth of *C. pseudodiphtheriticum* in vitro by an unknown mechanism [98], although it is not clear that the antagonism between these two species can have a positive impact on the host.

On the other hand, *C. propinquum*, a common nasal commensal bacterium that is closely related to *C. pseudodiphtheriticum*, releases siderophores that are able to sequester iron from the environment [87], this sequestration mechanism has an indirect impact on growth competitive CoNS species, including *S. epidermidis*.

### 5.3. Cutibacterium

Similar to *Corynebacterium*, *Cutibacterium* participates in antagonistic and symbiotic interactions with some species of Firmicutes. Some *Cutibacterium* species release coproporphyrin III which induces biofilm formation in *S. aureus* to promote nasal colonization [99]. In the case of *C. acnes*, it synthesizes and releases CAMP factor, which has a synergistic effect towards the hemolytic activity of *S. aureus* toxin β-hemolysin, thus helping the establishment of *S. aureus* within the host and promoting its invasiveness [93]. 

*S. epidermidis* can antagonize the effect of *C. acnes* by different mechanisms; the nasal cavity isolates of *S. epidermidis* can ferment glycerol, a common carbon source on the surface of human epithelial cells, converting it to succinic acid which is effective in limiting the growth of *C. acnes* [100]. Moreover, the physical contact interaction between *S. epidermidis* strain F21 and *C. acnes* has shown antimicrobial activity against *C. acnes* [101]. *S. epidermidis* FS1 has the presence in its genome of a putative lactococcin 972 superfamily bacteriocin, of a cognate immunity protein and of an epidermin-like peptide precursor that is proposed as responsible for its killing activity [101]. Similarly, *S. epidermidis* strain 14.1.R1, which possesses specific antimicrobial activity against *C. acnes*, possesses an ESAT-6 secretion system that may mediate the anti-*C. acnes* phenotype of this strain [101]. There are other mechanisms of inhibition for *C. acnes* by *S. epidermidis,* but they are shown to occur in the skin.

### 5.4. Moraxella catarrhalis

Beyond the events mentioned above, a competitive interaction between *S. epidermidis* and the opportunistic pathogen *M. catarrhalis* has been characterized. The result of this interaction is that some strains of *S. epidermidis* synthesize and excrete the compound nukacin, which has a direct effect on the death of *M. catarrhalis* [98].

### 5.5. Streptococcus pyogenes

Although *S. pyogenes* is not frequently found colonizing the nasal cavity, it has an antibiofilm activity against *S. aureus* with the production of the SpeB protease that is capable of breaking the *S. aureus*’ biofilm through the cleavage of SdrC from *S. aureus*. In contrast, *S. epidermidis* nasal isolates show in vitro strong inhibition on some *S. pyogenes* strains but with a yet unknown mechanism [98].

## 6. Other Mechanisms of Action of *S. epidermidis*

*S. epidermidis* produces and secretes antimicrobial peptides named bacteriocins that have antimicrobial activity [84,95,99]. Bacteriocin genes are generally encoded in mobile genetic elements such as plasmids and are rarely found in the bacterial genome [99]. In the nasal cavity, *S. epidermidis* bacteriocins kill *S. aureus* and other bacteria that cause UTR infections such as *D. pigrum* and *M. catarrhalis* [22,98].

As one of the functions of the microbiome of the nasal cavity is to enhance the immune system. In the case of *S. epidermidis,* it eliminates pathogenic bacteria by the activation and modulation of the innate immunity of the nasal epithelium. In homeostasis, *S. epidermidis* and the innate immune system collaborate to prevent UTR infections caused by viruses and bacteria [22,71]. The epithelial cells of the nose prevent the colonization of pathogenic bacteria by secretion of antimicrobial peptides (AMP) induced by innate immunity [102]. Lai Y, et al. (2010) [103], demonstrated that a small molecule (<10 kDa) produced and secreted by the reference strain *S. epidermidis* 1457, stimulates the expression of TLR-2-dependent β-human defensins (hBDs) 2 and 3 in normal human keratinocytes, which promote antimicrobial defense against bacterial skin infections. Quia Liu, et al. (2020) [22] treated normal human nasal epithelial cells (NHNE) with CFCM obtained from cultures of *S. epidermidis* from the nasal cavity and from a clinical origin and found the expression of two important AMPs: hBD3 and LL37. Similarly, the secretable products of *S. epidermidis* (commensal and clinical) stimulate the production of the AMPs LL37 and hBD3 in a more intense way compared to other bacteria such as *S. aureus, M. catarrhalis, C. propinquum*, and *D. pigrum*. In general, nasal cavity commensal strains of *S. epidermidis* induce higher AMPs than clinical strains.

Animal models of respiratory infection have demonstrated the role of the respiratory microbiota in the elimination of pathogens. The respiratory microbiota, like the intestinal microbiota, protect against respiratory infections caused by the main human pathogens such as *S. pneumoniae* and *Klebsiella pneumoniae*. Using a murine model, it was determined that *S. epidermidis* protects against respiratory infections (*S. pneumoniae* and *K. pneumoniae*) by the activation of Nod2 receptors present in the epithelial cells of the nasal cavity, and that event leads the epithelial cells to the production of IL-17A. IL-17A stimulates the production and release of granulocyte-macrophage colony-stimulating factor (GM-CSF) in epithelial cells, and as a consequence, alveolar macrophages are activated to kill and eliminate these pathogens [104].

All this evidence indicates the role of the microbiota and especially of *S. epidermidis* in the protection against bacterial infection in the human upper respiratory tract; however, despite such evidence, there are currently few studies on the use of microbiota, or specifically *S. epidermidis*, as therapeutics or probiotics for the treatment of bacterial infections in the respiratory tract. The direct use of nasal commensal microbes as probiotics that can eliminate the presence of opportunistic pathogens is a particularly intriguing approach. Such is the case of *C. pseudodiphtheriticum* and *S. epidermidis*, two potential candidates, which have been shown to reduce the growth and nasal colonization of *S. aureus* or *St. pneumoniae* [78,96]. Furthermore, the application of *C. pseudodiphtheriticum* to the nasal cavity of infant mice improved clinical outcomes of infection with a respiratory syncytial virus and *St. pneumoniae* via modulation of the host immune system [105]. These data suggest that the application of these bacteria as probiotics could be the alternative to prevent diseases.

## 7. *S. epidermidis* as a Regulator of Respiratory Viral Infections

Related to viral respiratory tract infections, *S. epidermidis* suppresses the infectivity of several influenza viruses [106]. In 2016 Chen HW, et al. demonstrated that the products secreted by commensal *S. epidermidis* strains ATCC12228 and ATCC1457 inhibit the hemagglutinating activity of influenza A and B viral strains (IVA, IVB), and also in clinical IVA strains such as H1N1 [107]. The secretable molecule responsible for the inhibition of the hemagglutinating activity of IVA or IVB is the giant extracellular matrix binding protein (Embp; 460 kDa) [107], since the mutant strain *S. epidermidis* Embp demonstrated a lack of inhibition of viral hemagglutination. In a model of embryonated eggs injected with rEmbp6599 and immediately infected with the reference strain IVA, the mortality of the embryos was reduced about 20% after one day post-infection and approximately 30% at day 8 post-infection [107]. In chickens inoculated intranasally with rEmbp6599 and infected with H6N1 virus, there was a limitation in the viral replication [107], in addition, the tissues of chickens inoculated with rEmbp6599 induced the expression of interleukin (IL)-6 and interferon-α (IFN-α) [107]. With these findings it is demonstrated that *S. epidermidis* and the purified protein Embp can be used as anti-influenza agents, not only to prevent viral invasion in humans but also to control the transmission between species of influenza viruses [107]. In another work, it was shown that isolates of staphylococci from species other than *S. epidermidis* coming from the nostrils of chickens, goats, cows and sheep are able to inhibit the hemagglutinating activity of New Castle virus and bovine virus parainfluenza virus type 3, indicating that the inhibition of hemagglutinating activity is not exclusive to the species *S. epidermidis* [106] and is a characteristic of CoNS. 

IFN-α, IFN-β, and IFN-λ constitute the first line of defense against microbial infections, mainly viral infections [108]. It was recently shown that among IFNs, IFN-λ is a critical immunomodulator against viral infections in the epithelial mucosa [109]. IFN-λ is considered to be responsible for the protection against viral invaders in the respiratory tract and plays an important role in local innate antiviral immunity [109,110]. In vitro studies and in animal models have shown that IFN-λ production and secretion is activated and modulated by commensal bacteria that reside in the nostrils [109]. Hyun K, et al. (2019) [109], used normal human nasal epithelium cells (NHNE) co-cultured with *S. epidermidis* from the nostrils of healthy subjects (8h co-culture) and inoculated with H1N1, and they observed the absence of viral infectivity, however, when the commensal strain *S. epidermidis* ATCC12228 was used, the antiviral protection was lost. The proposed mechanism of this viral inhibition is by the production of IFN-λ in NHNE cells induced by *S. epidermidis*, this effect occurs even in the absence of viral exposure. In a murine IVA infection model with a previous inoculation of *S. epidermidis*, it limited the spread of IVA to the lungs by stimulating innate immunity in which IFN-λ suppresses the replication of IVA in the nasal mucosa. [109].

## 8. Possible Involvement of *S. epidermidis* in COVID-19 Disease

So far it is not known whether *S. epidermidis* has an antagonistic effect with other viruses that affect the respiratory tract, and without a doubt, one with great current interest is the SARS-CoV-2 coronavirus (COVID-19). This virus belongs to the betacoronaviruses group and within this group, some viruses have a hemagglutinin-esterase, but SARS-CoV and SARS-CoV-2 do not [111]. The reports about viral inhibition induced by *S. epidermidis* were developed using influenza viruses that have hemagglutinating activity, and the mechanism of regulation of *S. epidermidis* is through the inhibition of viral hemagglutinin. On the other hand, although there are no data about the participation of the microbiota to inhibit SARS-CoV-2 in the nose, it is important to analyze the chance that the innate immunity of the respiratory epithelium could be activated with the use of *S. epidermidis*, mainly in the production of IFN-λ, as occurs with IVA, to induce the expression of antiviral genes of the interferon pathway.

When an infection occurs in the nasal cavity, it generates dysbiosis, as in the case of SARS-CoV-2. In the nose the ciliated cells are the primary target cells for the SARS-CoV-2 infection, it replicates and releases viral particles. Infected cells lose their cilia, losing their mucociliary capacity and favoring the infection. At the beginning of the infection, there is a fight to counteract the infection by SARSCoV-2, where the innate immune system and the nasal cells participate strongly [112], however, the role that the microbiota of the nasal cavity on SARS-CoV-2 remains unknown.

It has been established that children are less susceptible to SARS-CoV-2, and if they become infected, they suffer milder illness compared to adults, who are more susceptible and have a serious illness. In a microbiota analysis study in children, adolescents and young adults infected with SARS-CoV-2, higher abundances of *Corynebacterium* species were associated with SARS-CoV-2 infection with respiratory symptoms, while higher abundances of *D. pigrum* were present in SARS-CoV-2-infected individuals without respiratory symptoms. Besides, the abundance of these bacteria was strongly associated with age [113]. In the comparison between COVID-19-positive and negative patients, significant changes in the microbiota were found with higher abundance of Propionibacteriaceae and a reduction in the abundance of *C. accolens* in negative patients. In the cases of affected patients there was a significant increase in the abundance of Proteobacteria and a decrease in Fusobacteria and Bacteroidetes, as well as an increase in the abundance of opportunistic pathogens such as *Haemophilus*, *Stenotrophomonas*, *Acineobacter*, *Moraxella*, *Corynebacterium*, *Gemella*, *Ralstonia*, and *Pseudomonas* [114].

The age and sex of the patients are potential factors for the COVID-19 infection related to the enrichment of specific bacterial communities in the upper respiratory tract, for example, in the age of 31-45 years, higher abundance of *Haemophilus*, *Stenotrophomonas*, *Leptotrichia*, *Acinetobacter*, *Fusobacterium*, *Prevotella*, *Pseudomonas*, *Staphylococcus*, and *Lachnoaerobaculum* are present [115]. Another study indicated that dysbiosis occurs in severely ill patients with predominant respiratory microbial taxa of *Burkholderia cepacia* complex (BCC), *S. epidermidis*, and *Mycoplasma* spp. (including *M. hominis* and *M. orale*) [116]. On the other hand, in ventilator-associated complications (VACs) in COVID-19 patients, *C. pseudodiphtheriticum* and *C. striatum* are pathogens found in the lower respiratory tract infections, reported in three cases of VAC due to *C. pseudodiphtheriticum* in COVID-19 patients [117]. 

As it is known, SARS-CoV-2 uses angiotensin-converting enzyme 2 (ACE2) as its receptor for internalization to target cells, and the binding affinity of the spike (S) protein of SARS-CoV-2 to ACE2 is decisive for infection [118,119,120,121]. Host proteases are involved in cellular invasion by SARS-CoV-2, and the transmembrane serine protease 2 (TMPRSS2) is the main host protease that leads to the breakdown of SARS-CoV-2 S protein in the nasal epithelial cells [118]. Unlike other viral receptors, the presence of ACE2 is higher in human nasal mucosa than in lung tissue cells, and at the cellular level, the expression of ACE2 mRNA is higher in Normal human nasal epithelial (NHNE) than in normal human bronchial epithelial (NHBE) cells. Regarding the expression of TMPRSS2 mRNA, it occurs opposite to ACE2, that is, lower in human nasal mucosa and NHNE cells than in lung and NHBE cells, with some specificity to suprabasal cells. Regarding *S. epidermidis*, the effect of SARS-CoV-2 on its relative abundance in patients is not known in detail. In a recent study performed by Ji JY, et al. (2021) [122] the role of *S. epidermidis* in SARS-CoV-2 infection was analyzed. In this study NHNE cells from healthy subjects were inoculated with *S. epidermidis*, and the expression of ACE2 and TMPRSS2 decreased mainly in the basal NHNE cells in response to *S. epidermidis*; on the contrary, the expression of TMRPSS2 was elevated in suprabasal NHNE cells, and conversely, its expression was reduced in multicilliated and basal NHNE cells. In healthy subjects, it was found that those with a higher number of *S. epidermidis* in the nasal mucosa have a low expression of ACE2 and TMRPSS2, and it is the opposite for those healthy subjects with a low amount of *S. epidermidis*. In the case of NHNE cells, they also reduced the expression of ACE2 and TMRPSS2 when they are in the presence of *S. aureus*, but not with *C. pseudodiphtheriticum* or influenza A virus [122]. These results clearly demonstrate that the presence of *S. epidermidis* or of other *Staphylococcus* in the nasal cavity has the effect to reduce significantly the expression of receptors and proteases involved in SARS-CoV-2 infection, suggesting important participation of *S. epidermidis* in the control of this virus infection.

Regarding interferons (IFNs), they play a crucial role in the immune response against viral infections. Type I IFNs (IFN-α and IFN-β) are recognized by specific receptors expressed on the cell surface, and this binding activates the JAK-STAT signaling pathway that leads to the up-regulation of numerous IFN stimulated genes (ISGs) with antiviral activity. The IFN-λ family (also known as type III interferons) [108,123] are recognized by a different receptor (IFN-λR1) but also activate the JAK-STAT signaling pathway. IFN-λ provides the first line of immunological defense against viral infections of the respiratory tract [108,123], such as influenza viruses. Recombinant IFN-λ intranasal treatment inhibits influenza virus replication, protects the upper airways, and blocks virus transmission to uninfected mice [123]. Related to the MERS-CoV strain virus (human coronavirus EMC) and SARS-CoV, they do not induce the expression of type I and III IFNs in respiratory tissue culture [123], and in the case of SARS-CoV-2 the expression of IFN-λ is especially weak in an ex vivo lung tissue model, suggesting that IFN-λ might be particularly effective against SARS-CoV-2.

In macaque monkeys infected with SARS-CoV and treated prophylactically with intramuscular pegylated IFN-α, the viral replication and excretion, as well as pulmonary damage are reduced [123]. In a human airway epithelial cell culture model, IFN-λ3 and IFN-λ4 caused antiviral effects to MERS-CoV [123]. These studies have shown that the timing of IFN administration is critical for the treatment, as late treatment with IFN-λ may not have a beneficial effect [123]. Although there are still no results with treatments against SARS-CoV-2 using IFN-λ, with these data it can be proposed that IFN-λ would have prophylactic protection against coronavirus infections. In the case of SARS-CoV-2, preclinical data from various animal model studies suggest that pegylated IFN-λ1 might reduce the disease severity and risk of transmission [123]. While more specific measures are being developed, pegylated IFN-λ1 should be evaluated as part of an early and rapid response to attenuate disease and prevent infection spread [123]. The pegylated IFN-λ1 has not been tested in patients with respiratory infections and, ideally, should be first studied in patients with early SARS-CoV-2 infection or as prophylaxis [123].

The expression of interferons has been studied in COVID-19 patients and it was shown that in mildly symptomatic patients there is a correlation between the presence of nasal IFN I/III and the symptoms of the disease. The patients showed a high peak of IFN I/III expression at the onset of symptoms and returned to baseline since day 10. In cases of critically ill COVID-19 patients, a low nasal expression of IFN I/III is accompanied by a high viral load due to the presence of autoantibodies against IFN-I in the nasopharyngeal mucus and in blood [124]. 

When studying the immune response against SARS-CoV-2 in child and adult patients, it was shown that in the nasal mucosa there is a low proportion of immune and epithelial cells in older subjects and a high proportion in younger subjects [125], explaining the fact that adult patients are the most susceptible group to COVID-19. This also occurs in the microbiota of the nasal cavity, where there is a low microbial load (in particular with *S. epidermidis*) in advanced-age people. SARS-CoV-2 induces low levels of expression of genes associated with the interferon I/III pathway and instead induces the expression of pro-inflammatory chemokines and cytokines (such as IL-6) that are responsible for the clinical manifestations of the disease [119]. In some patients with severe COVID-19 [126], defects in the response to interferon type I were found due to rare genetic variants. These findings support the idea that SARS-CoV-2 attenuates the main immune response characterized by type I and type III airway interferons against respiratory viruses.

**Figure 2:**
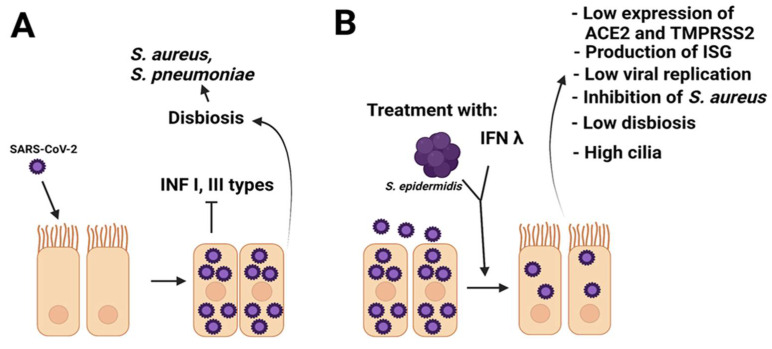
COVID-19 treatment proposal. (**A**) The SARS-CoV-2 virus infects the epithelial cells of the nasal cavity and replicates. Viral infection causes low production of IFN I/III and dysbiosis with a relative abundance of opportunistic pathogens. (**B**) Direct application of nasal cavity with commensal *S. epidermidis* and IFN λ at the beginning of the infection with SARS-CoV-2 could help to improve and restore the immune response and the healthy microbiota. ISG: interferon-induced genes.

Based on the above, we propose that early IFN therapies can be used together with *S. epidermidis* transfers to counteract SARS-CoV-2 infection (Figure 2). The early administration of IFN therapies and *S. epidermidis* would be a key factor since it is known that the delayed type I response to interferon leads to the accumulation of inflammatory monocytes and macrophages that co-help with severe immunopathology in mice infected with SARS-CoV [127].

On the other hand, Table 1 shows evidence of the probiotic role of *S. epidermidis* in the protection against viral and bacterial infection in the upper respiratory tract; however, despite such evidence, there are currently few studies on the use of microbiota, or specifically *S. epidermidis*, as therapeutics or probiotics for the treatment of viral and bacterial infections in the respiratory tract. Currently, the strongest evidence on the potential of *S. epidermidis* as a probiotic has been shown for the treatment of some inflammatory skin diseases, e.g., atopic dermatitis, or skin infections [85,103]. Moreover, *S. epidermidis* can restore impaired collagen in the dermal extracellular matrix, providing integrity and elasticity to the skin by the mechanism FFaR2/p-ERK signaling [128]. The evidence of *S. epidermidis* in the protection against viral and bacterial infection in the upper respiratory tract is described below.

## 9. Conclusions

SARS-CoV-2 induces dysbiosis towards opportunistic pathogenic bacteria and, on the contrary, *S. epidermidis* has an inhibitory effect on these bacteria, and also *S. epidermidis* induces low expression of the receptor ACE2 and protease TMRPSS2 in epithelial cells. In addition, *S. epidermidis* aids the induction and maintenance of IFNs expression in the epithelial cells of the nasal cavity, contributing to the immune response against SARS-CoV-2. Therefore, we suggest that the direct inoculation of the nasal cavity with commensal *S. epidermidis* as a probiotic can help to reduce the infection of SARS-CoV-2, and together with vaccines and anti-COVID-19 drugs, it could be possible to control or eliminate the pandemic of COVID-19. 

## Figures and Tables

**Table 1:** Studies carried out in humans and animal models where the probiotic potential of *S. epidermidis* was demonstrated.

Strains or Products/Reference	Pathogen	Pre-Clinical Studies on Human and Animal Models	Main Findings
*S. epidermidis* (ST9-N442); commensal, [22].	*S. epidermidis* (ST2); infection. *S. aureus*; infection.	Murine model of Respiratory Tract Infection (RTI), first pre-colonization intranasally with *S. epidermidis* (ST9-N442) or pathogens.	*S. epidermidis* (ST9-N44-2) increase more expresión of CRAMP. *S. epidermidis* (ST9-N44-2) efficiently outcompeted the two pathogenic bacteria *S. aureus* and *M. catarrhalis* in vivo and led to decreased signs of infection caused by these pathogens.
	*M. catarrhalis*; infection.	Murine model RTI co-colonized intranasally with *S. epidermidis* (ST9-N442) and pathogens.	
*S. epidermidis* NRS122, [73].	*S. aureus* BD02-31	Murine model RTI pre-colonized intranasally with *S. epidermidis* NRS122 followed by intranasally challenge with *S. aureus* BD02-31.	Pre-colonization of mouse nares with *S. epidermidis* NRS122 reduces colonization of *S. aureus* BD02-31.
*S. epidermidis* (wild type, JK16); Esp-positive.	*S. aureus*	In a pilot sudy, *S. epidermidis* cells or purified Esp were introduced into anterior nares of volunters who were *S. aureus* carriers.	*S. epidermidis* (JK16) and purified Esp eliminated *S.aureus* colonization. *S. epidermidis* Esp-deficient and *S. epidermidis* (JK11) did not reduce colonization of *S. aureus.*
*S. epidermidis* Esp-deficient.			
*S. epidermidis* (JK11); *Esp*-negative.			
Purified Esp, [78].			
*S. epidermidis* AMT-A9, [85].	*S. aureus*	In a pilot study, *S. epidermidis* AMT-A9 was inoculated into wound atopic dermatitis (AD) of volunteers who were *S. aureus* carriers.	*S. epidermidis* AMT-A9 eliminated *S. aureus* from the wound (AD), and clinical manifestation of AD improved.
*S. epidermidis* 1457, [103]	Group A *Streptococcus* (GAS)	Murine skin infection model with GAS treated with *S. epidermidis* 1457.	*S. epidermidis* 1457 protects mice against GAS by the activation of TLR2 and induction of hBDs 2 y 3.
*S. epidermidis* (human), [104].	*S. pneumoniae*	Murine model RTI pre-colonized intranasally with *S. epidermidis* followed by intranasal challenge with *S. pneumoniae* or *K. pneumoniae*.	Pre-colonization of mouse nares with *S. epidermidis* limited the spread of *S. pneumoniae* and *K. pneumoniae* by the activation of Nod2 receptor, production of IL-17A, release of GM-CSF, and activation of alveolar macrophages.
	*K. pneumoniae*		
rEMbp6599 of *S. epidermidis,* [107].	IVA	Chicken model of RTI pre-colonized intranasally with rEMbp6599 followed by intranasal challenge with IVA.	rEMbp6599 protects against RTI (IVA) by reducing the tissue viral load and inducing robust expression of antiviral cytokines (IFN-α, IL-6, and Mx)
*S. epidermidis* (human), [109].	IVA	Murine model of RTI pre-colonized with *S. epidermidis* followed by intransal challenge with IVA	Pre-colonization of mouse nares with *S. epidermidis* limited the spread of IVA to the lungs by stimulating innate immunity in which IFN-λ suppresses the replication of IVA in the nasal mucosa.

ST, Sequence Type; CRAMP, mouse cathelicidin-related Antimicrobial Peptide; Esp, *Staphylococcus epidermidis* protease; hBDs, Human β-defensin; GM-CSF, granulocyte-macrophage colony-stimulating factor; rEMbp6599, recombinant giant extracellular matrix-binding protein of *S. epidermidis*; IVA, Avian Influenza virus A.

## Data Availability

Not applicable.
